# Very severe immune thrombocytopenia following SARS-CoV-2 vaccination requiring splenectomy: a case report

**DOI:** 10.1186/s12959-022-00404-z

**Published:** 2022-08-23

**Authors:** Marc Weiner, Robert Rodriguez-Vigouroux, Stavroula Masouridi-Levrat, Kaveh Samii

**Affiliations:** 1 Department of Oncology, Division of Oncology, Geneva University Hospital, Rue Gabrielle-Perret-Gentil-4, 1205, Geneva, Switzerland; 2 Department of Medicine, Division of General Internal Medicine, Geneva University Hospital, Rue Gabrielle-Perret-Gentil-4, 1205, Geneva, Switzerland; 3 Department of Oncology, Division of Hematology, Geneva University Hospital, Rue Gabrielle-Perret-Gentil-4, 1205, Geneva, Switzerland

**Keywords:** COVID-19, Vaccine, Immune thrombocytopenia, SARS-CoV-2, Splenectomy, Case report

## Abstract

**Background:**

Some conventional vaccines have been recognized as a cause of secondary immune thrombocytopenia (ITP). According to recent publications, mRNA vaccines are probably associated with an increased risk of ITP.

**Case presentation:**

Our patient developed severe ITP one week after the second dose of COVID-19 mRNA vaccine. Medical management was not effective, requiring splenectomy to obtain sustained remission.

**Conclusion:**

Considering the temporality and immunological hypothesis, we consider the vaccine to be the trigger of this ITP. To our knowledge, our case is, to date, the most severe case of ITP reported following SARS-CoV-2 vaccination and could help for the therapeutic management of similar patients.

## Background

Immune thrombocytopenia (ITP) is an autoimmune disorder characterized by increased platelet destruction [1] that could result in severe bleeding. It involves elements of the innate and adaptive immune systems. It is an exclusion diagnosis suspected in patients with a platelet count < 100 G/L [2]. It is defined as secondary when a cause is identified (infections, auto-immune diseases, lymphoproliferative disorders, drugs and vaccines [3] or as primary, meaning idiopathic. The goal of its management is to restore a sufficient platelet level to prevent hemorrhagic complications. Actual treatment options include immunosuppressive drugs, thrombopoietin agonists and intravenous immunoglobulins. Splenectomy was the most frequent second-line treatment in steroid-refractory ITP patients before more therapeutic options became available [4]. In our centre, splenectomy is now usually reserved as a therapy of last resort when medical treatments have failed, due to the inherent risks associated with surgery.

We present the case of a 42 year-old man with a very severe ITP in the context of his SARS-CoV-2 (Severe Acute Respiratory Syndrome-Coronavirus-2) mRNA vaccination.

## Case presentation

### First hospitalization (D1 to D5)

We report the case of a 42 year-old Caucasian male with a history of harmful alcohol consumption (4 standard units a day) and allergic asthma who was evaluated in our emergency department complaining of spontaneous hematomas on the limbs as well as cutaneous and mucosal petechiae. He also reported one episode of self-limiting epistaxis. He denied any blood in his stools or hematuria and was not taking any medication. He had quit smoking 5 years ago (20 pack-year) and currently vapes. Family history was notable for a brother with scleroderma.

He reported having received his second dose of the COVID-19 (coronavirus disease 2019) vaccine elasomeran one week prior to noticing bruises on his limbs.

Initial evaluation revealed hematomas on all limbs and petechiae with a hemorrhagic bulla on the inside of his cheeks. No hepatosplenomegaly or adenomegaly was noted either on physical examination or on abdominal ultrasound.

Laboratory workup revealed normal hemoglobin level and leukocyte count but severe thrombocytopenia with 2 G/l (150–300 G/l). Peripheral blood smear confirmed true thrombocytopenia without any other abnormality. Coagulation profile, renal and liver function tests were all within normal range. Lactate dehydrogenase reached 390 U/l (87–210 U/l). Protein electrophoresis and immunofixation excluded any paraprotein. A nasopharyngeal swab for SARS-CoV-2 was negative and serology showed a response to vaccination without prior exposure to the virus (anti-S positive and anti-N negative).

Vitamin B12 and B9 levels were low: cyanocobalamin 105 pmol/l (125–574 pmol/l) and folate 4.5 nmol/l (8–60 nmol/l). Normal levels of intrinsic factor excluded pernicious anemia, and after vitamin supplementation, the values normalized within two weeks. Ferritin was slightly elevated at 449 µg/l (11–342 µg/l).

Hepatitis B, C and HIV were excluded as well as acute Cytomegalovirus, Epstein-Barr virus and Parvovirus B19 infections.

**Fig. 1:**
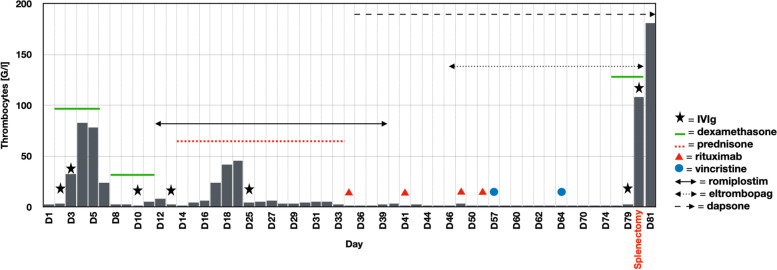
Evolution of the patient’s platelet level after different treatments. The extreme values following splenectomy were not included to avoid scale distortion

As a result of this workup, ITP was immediately suspected. We excluded vitamin B12 and folate deficiencies as the cause of thrombocytopenia since there was no anemia or macrocytosis and no improvement after vitamin supplementation. Treatment commenced with oral dexamethasone at 40 mg/day on D2 to D5 in combination with intravenous immunoglobulins (IVIg) at 1 g/kg on D2 and D3, according to the hemorrhagic score (9 points) [5]. Response was considered satisfactory with thrombocyte levels reaching 78 G/l on D5 (Fig. 1), allowing patient’s discharge with close ambulatory follow-up.

### Second hospitalization (D8 to D19)

Follow-up consultation 3 days after discharge revealed relapse of thrombocytopenia to 2 G/l, requiring a second hospitalization for the same treatment with oral dexamethasone (40 mg/day) from D8 to D11 and IVIg (1 g/kg) on D10 and D13. As this combination now proved ineffective, treatment with romiplostim was initiated at 7 µg/kg/week (= 500 µg/week) on D12, increased to 10 µg/kg/week (= 700 µg/week) as of D19 because no improvement was noticed.

Immunologic work-up showed a slightly increased level of anti-SSA antibodies (52KDa) at 26 units (normal: < 20 units). In the absence of any clinical manifestation compatible with an underlying auto-immune disease, this result was considered irrelevant.

Prednisone 1 mg/kg/day for 3 weeks was initiated on D14. These combined treatments increased platelet levels to 45 G/l, allowing patient’s discharge on D19.

### Third hospitalization (D25 to D88)

Six days later, during ambulatory follow-up, the patient had again relapsed (thrombocytes = 2 G/l), and was re-admitted to the hospital. He received one dose of IVIg (1 g/kg) upon admission (D25). The treatments with romiplostim and prednisone continued without improving platelet levels, which stagnated below 10 G/l. After 3 weeks prednisone was slowly tapered.

Given the known association of *Helicobacter pylori* infection with ITP, we performed a serology which came back positive. The patient received 10 days of an eradication regimen consisting of amoxicilline, clarithromycine, metronidazole and esomeprazole. A negative breath test one month later confirmed eradication.

Peripheral blood flow cytometry did not show any abnormal lymphocyte population.

**Fig. 2:**
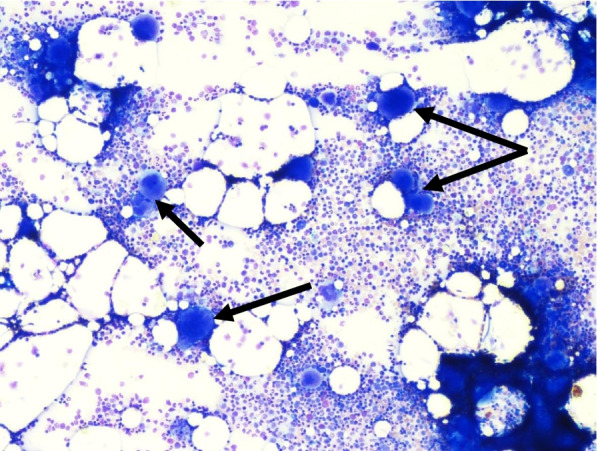
Smear of the patient’s bone marrow showing numerous normal megakaryocytes (arrows)

Bone marrow aspirate was compatible with ITP, showing elevated numbers of megakaryocytes without dysplasia and no blasts on the bone marrow smear nor on flow cytometry (see Fig. 2). Circulating anti-platelet antibodies against glycoprotein Ia-IIa were detected, demonstrating a humoral auto-immunity in the disease.

Despite all these treatments, profound thrombocytopenia persisted. A treatment of weekly rituximab for 4 weeks at the dose of 375 mg/m^2^ (700 mg/week) was started on D34 after a negative interferon-gamma release assay (QuantiFERON). After one month of romiplostim at the maximum dose, we switched on D46 to eltrombopag 75 mg/day.

On D36, we initiated a treatment with dapsone 100 mg/day, as both a treatment option for ITP and an approved *Pneumocystis jirovecii* prophylaxis, indicated in the context of drug-induced immunosuppression. In order to offer some bleeding protection, 500 mg of oral tranexamic acid was administered daily, being the minimal effective dose in our patient.

In the absence of improvement, it was decided to administer vinca alkaloids due to their rapid onset of action. The patient received 2 doses of vincristine, 2 mg each, on D57 and D64. Unfortunately, platelet levels remained at 1 G/l two weeks later.

Since diverse medical therapies had failed, and the patient remained at high risk for major bleeding, splenectomy was planned. In order to establish a sufficient platelet level for surgery, the patient received another 4 days (D76 to D80) of dexamethasone 40 mg/day and 2 days of IVIg (1 g/kg) on D79 and D80. This induced a rapid rise in platelet levels to 181 G/l. Treatment with eltrombopag was abruptly discontinued.

The patient underwent laparoscopic splenectomy on D80. Platelet levels then rapidly rose, reaching 1,520 G/l on D87, and progressively normalized. Six months later, the patient’s platelet levels remained in the normal range (302 G/l on D259).

## Discussion

The SARS-CoV-2 vaccination program can be considered the largest worldwide vaccination campaign in history. Western countries have vaccinated a majority of their populations with two new mRNA vaccines: tozinameran and elasomeran.

Secondary ITP has been reported after the administration of several vaccines but an association has been confirmed for only measles-mumps-rubella vaccines [6]. The assumed mechanism is the triggering of an immune response targeting platelets possibly via molecular mimicry. As mRNA vaccines differ from conventional vaccines and have been used only recently for the prevention of COVID-19, the mechanism that could explain a potential association with ITP remains unknown. Thrombocytopenia is often reported in the course of COVID-19 disease and is considered a marker of poor prognosis [7]. To our knowledge, no molecular mimicry has been observed between SARS-CoV-2 components and platelet epitopes. However, unpublished research suggests that molecular mimicry could occur between the Spike protein of SARS-CoV-2 (of which translation is induced by mRNA vaccines) and human thrombopoietin [8].

The toll-like receptor 7, which recognizes single-stranded viral RNA, has been shown to be strongly activated after COVID-19 infection or mRNA vaccination. Interestingly, toll-like receptor 7 signaling participates in the immune pathogenesis of ITP [1].

From an epidemiological point of view, it has not yet been established if the occurrence of ITP following mRNA vaccination is coincidental or causally related [9]. Observation that the number of cases of ITP reported to the Vaccine Adverse Event Reporting System following vaccination does not exceed its incidence among the general population speaks against a causative relation [10] (1—4 per 100,000 persons in Europe and 9.5 per 100,000 persons in the United States of America, without seasonal variability [11].

Though first-line treatment with corticosteroids and IVIg is the standard of care for severe cases [2], other treatment modalities have not been clearly established. Some authors discourage the use of rituximab as a second-line agent because of its slow onset of action (ranging from 1 to 3 months after starting the treatment) and its negative impact on the humoral protection conferred by the vaccine [9]. Lee et al. suggest first adding a thrombopoietic agent and, if necessary, administering vinca alkaloids for their quicker onset of action (7 to 14 days [2, 12].

## Conclusion

To our knowledge, our case is the most severe ITP reported following SARS-CoV-2 vaccination with mRNA vaccines. One would be tempted to qualify it as refractory ITP due to its resistance to multiple therapeutic modalities, but this definition would also require resistance to splenectomy, which ultimately proved effective. The temporal correlation and possible immunological pathways provide some evidence to support a causal link to the vaccine. Continued pharmacovigilance is necessary to clearly establish whether or not ITP may be caused by mRNA vaccines in order to manage the potential risk.

## Data Availability

Data sharing is not applicable to this article as no datasets were generated or analyzed during the current study.

## Abbreviations

ITP: Immune thrombocytopenia
COVID-19: Coronavirus disease 2019
mRNA: Messenger ribonucleic acid
SARS-CoV-2: Severe Acute Respiratory Syndrome-Coronavirus-2
IVIg: Intravenous immunoglobulins
